# Exercise Preconditioning Protects against Spinal Cord Injury in Rats by Upregulating Neuronal and Astroglial Heat Shock Protein 72

**DOI:** 10.3390/ijms151019018

**Published:** 2014-10-20

**Authors:** Cheng-Kuei Chang, Willy Chou, Hung-Jung Lin, Yi-Ching Huang, Ling-Yu Tang, Mao-Tsun Lin, Ching-Ping Chang

**Affiliations:** 1 Graduate Institute of Injury Prevention and Control, Taipei Medical University, Taipei 110, Taiwan; E-Mail: chengkuei.chang@gmail.com; 2 Neurosurgical Department, Taipei Medical University Shuang-Ho Hospital, Taipei 235, Taiwan; 3 Physical Medicine and Rehabilitation Department, Chi Mei Medical Center, Tainan 710, Taiwan; E-Mail: ufan0101@ms22.hinet.net; 4 Department of Leisure Management, Cha Nan University of Pharmacy and Science, Tainan 717, Taiwan; 5 Department of Biotechnology, Southern Taiwan University of Science and Technology, Tainan 710, Taiwan; E-Mails: hjlin.cm@msa.hinet.net (H.-J.L.); sunyu0408@yahoo.com.tw (L.-Y.T.); 6 Department of Emergency Medicine, Chi Mei Medical Center, Tainan 710, Taiwan; 7 Department of Radiology, Chi Mei Medical Center, Liouying, Tainan 736, Taiwan; E-Mail: yichingh@mail2000.com.tw; 8 Department of Medical Research, Chi Mei Medical Center, Tainan 710, Taiwan

**Keywords:** spinal cord compression, neurological severity score, heat shock protein 72, exercise preconditioning

## Abstract

The heat shock protein 72 (HSP 72) is a universal marker of stress protein whose expression can be induced by physical exercise. Here we report that, in a localized model of spinal cord injury (SCI), exercised rats (given pre-SCI exercise) had significantly higher levels of neuronal and astroglial HSP 72, a lower functional deficit, fewer spinal cord contusions, and fewer apoptotic cells than did non-exercised rats. pSUPER plasmid expressing HSP 72 small interfering RNA (SiRNA-HSP 72) was injected into the injured spinal cords. In addition to reducing neuronal and astroglial HSP 72, the (SiRNA-HSP 72) significantly attenuated the beneficial effects of exercise preconditioning in reducing functional deficits as well as spinal cord contusion and apoptosis. Because exercise preconditioning induces increased neuronal and astroglial levels of HSP 72 in the gray matter of normal spinal cord tissue, exercise preconditioning promoted functional recovery in rats after SCI by upregulating neuronal and astroglial HSP 72 in the gray matter of the injured spinal cord. We reveal an important function of neuronal and astroglial HSP 72 in protecting neuronal and astroglial apoptosis in the injured spinal cord. We conclude that HSP 72-mediated exercise preconditioning is a promising strategy for facilitating functional recovery from SCI.

## 1. Introduction

In the heat shock protein (HSP) 70 family, HSP 72, the inducible isoform of HSP70, can be readily induced in the cytoplasm and nucleus of cells that are exposed to sublethal stressors, including exercise [1]. HSP 72 functions include inducing molecular chaperones [1], endogenous anti-apoptosis modulators [2], and anti-inflammatory agents [3]; controlling cell signaling [4] and modulating the immune reaction [5]; and by facilitating immunological responses to protect cells against pathogenic and other types of tissue damage [5]. Exercise increases serum HSP 72 in humans [6]. Physical exercise also increases HSP 72 in the vital organs of rats: the brain, heart, lungs, liver, and muscles [7,8,9]. To our knowledge, there are no published reports on the effects of exercise preconditioning or a spinal cord injury (SCI) on neuronal and glial levels of HSP 72 in the spinal cord.

A traumatic SCI is a lesion of neural elements of the spinal cord that can result in any degree of sensory and motor deficit, autonomic nervous system dysfunction, or bowel dysfunction [10]. Compared with non-exercised groups of animals, groups of treadmill-exercised animals showed greater functional recovery [11,12,13]. Exercise preconditioning (EP) increases HSP 72 expression, and the presence of HSP 72 before a stroke [9] or heatstroke [7] attenuates neuronal injury in rats. Again, it is not known whether a traumatic injury affects the neuronal or glial expression of HSP 72 in the injured spinal cord. Furthermore, it remains unclear whether functional recovery after an SCI and neuronal or glial expression of HSP 72 in the injured spinal cord can be affected by EP. To better understand exercise-based neuroprotection in SCI, we explain the effects of EP and SCI on the neuronal and glial expression of HSP 72 in the spinal cord of rats. Secondly, we describe the contributions of neuronal and glial expression of HSP 72 to EP mediated neuronal and glial survival in a rat model of SCI. In the central nervous system, topical injections of naked small interfering RNAs (siRNAs) [14,15,16,17] have been used successfully to induce gene silencing [15]. The pSUPER RNAί System (Oligo Engine, Seattle, WA, USA) efficiently and specifically downregulates gene expression [18], which functionally inactivates targeted genes. To inhibit HSP 72 expression in injured spinal cord tissue, the injured spinal cords were intraoperatively microinjected with pSUPER plasmid expressing HSP 72 small interfering RNA (siRNA-HSP 72).

## 2. Results

### 2.1. Spinal Cord HSP 72 Was Higher in Exercise-Preconditioned (EP^+^) Rats

**Figure 1:**
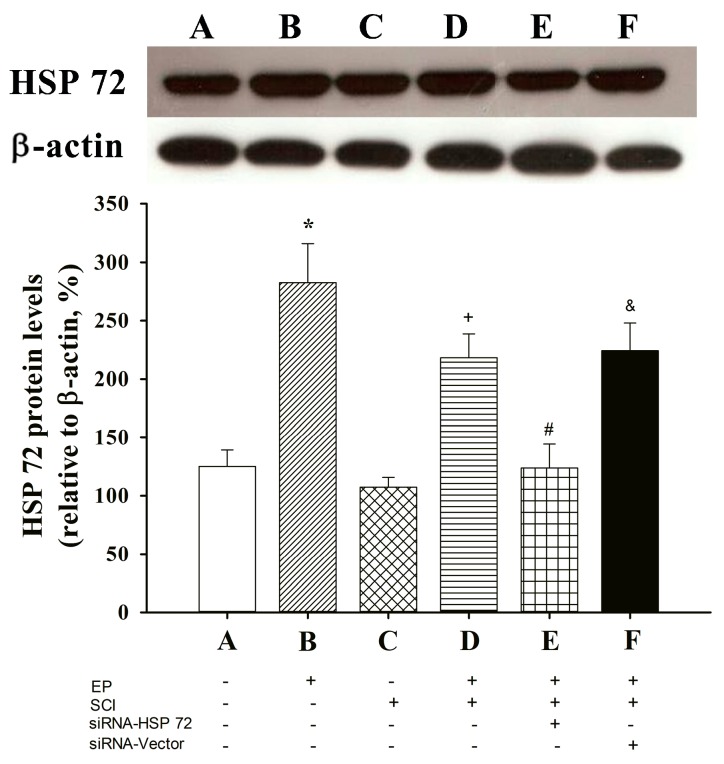
Effect of exercise on spinal cord expression of HSP 72 in different groups of rats. A: EP^−^ control; B: EP^+^ control; C: EP^−^ + SCI; D: EP^+^ + SCI; E: EP^+^ + siRNA-HSP 72 + SCI; F: EP^+^ + siRNA-Vector + SCI. Please see Experimental groups and procedures for the explanations of the abbreviations. Spinal cord expression of HSP 72 was assessed by western blot analysis seven days after injury or sham operation. The gels presented are representative of results from three separate experiments. Densitometry readings of gel bands expressed as arbitrary units of relative intensities to that of non-Exe + sham control. Values represent mean ± SD of three separate experiments. * *p* < 0.01 for B or D *vs.* A or C; ^+^ *p* < 0.05 for D *vs.* C; ^#^ *p* < 0.05 E *vs.* D; ^&^ *p* < 0.05 for F *vs.* E.

**Figure 2:**
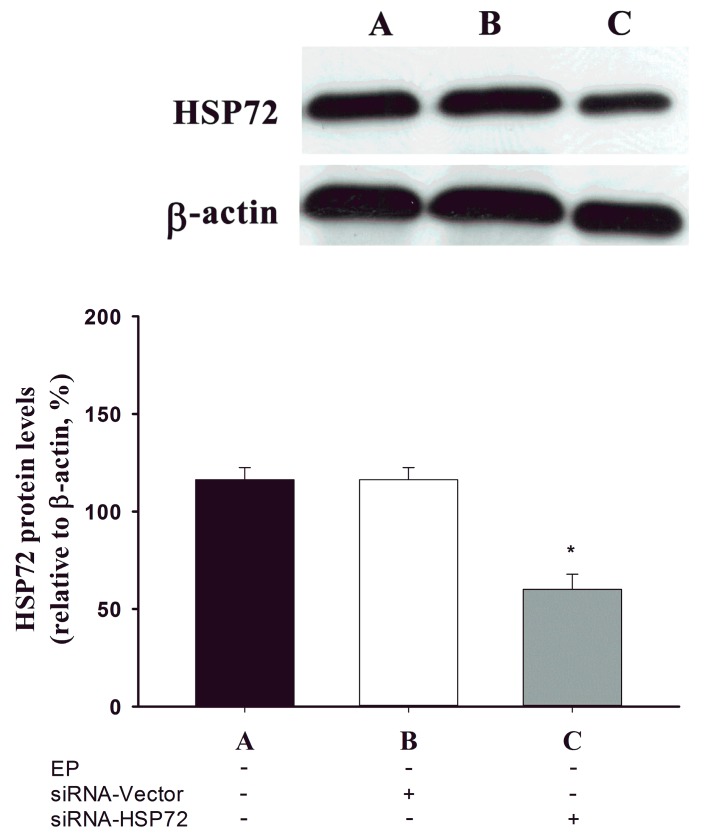
Effect of siRNA-HSP 72 on spinal cord expression of HSP 72 in different groups of rats. A: EP^−^ control; B: EP^−^ + siRNA-vector; C: EP^−^ + siRNA-HSP 72. Spinal cord expression of HSP 72 was assessed by Western blot analysis 7 days after injection in sham groups of rats with no exercise-preconditioning. The gels presented are representative of results from three separate experiments. Densitometry readings of gel bands represent mean ± SD of three separate experiments. * *p* < 0.05 for A *vs.* C.

Western blotting was used to analyze tissue samples from the spinal cords of all 6 groups of rats 7 days post-laminectomy. In the exercise-preconditioned rats (EP^+^ Control; *i.e.*, exercise preconditioned but not compression-induced SCI), levels of thoracic spinal cord HSP 72 were significantly higher than those for the not exercise-preconditioned rats (EP^−^ Control; *i.e.*, no exercise preconditioning and no compression-induced SCI), (*p* < 0.05, B *vs.* A) (Figure 1). In contrast, no differences were found in the spinal cord levels of HSP 72 between the EP^−^ + SCI group and the EP^−^ Control group (*p* > 0.05, C *vs.* A) (Figure 1). The EP^+^ + SCI group had significantly higher spinal cord levels of HSP 72 (*p <* 0.05, D *vs.* C) (Figure 1) than did the EP^−^ + SCI group. The spinal cord levels of HSP 72 were significantly lower in the EP^+^ + siRNA-HSP 72 + SCI group (*p <* 0.05, E *vs.* D) (Figure 1) than in the siRNA-vector group (*p* > 0.05, F *vs.* D) (Figure 1). Figure 2 showed that the protein levels of HSP 72 in the absence of exercise (EP^−^ + sham) were significantly decreased by siRNA-HSP 72 (*p* < 0.05), but not by siRNA-vector (*p* > 0.05).

### 2.2. Functional Recovery after SCI

**Figure 3:**
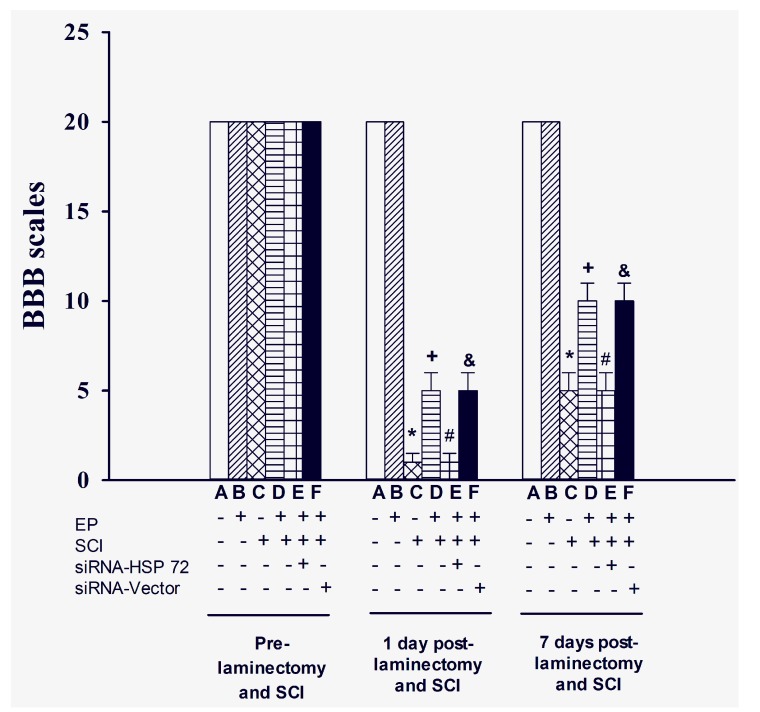
Effect of exercise on functional recovery following SCI. Data represent mean ± SD (*n* = 8 per group). * *p* < 0.001 for C group *vs.* A group; ^+^ *p* < 0.05 for D group *vs.* C group; ^#^ *p* < 0.05 for E group *vs.* D group; ^&^ *p* < 0.05 for F group *vs.* E group. Please see the legends of Figure 1 for the explanations of the group abbreviations.

Hind-limb functional recovery was assessed using BBB behavioral testing [19] 1 day before SCI, 1 day post-SCI, and for 7 successive days. The lower BBB scores indicate more severe hind-limb dysfunction. BBB scores post-SCI were significantly higher in the EP^+^ + SCI group than in the EP^−^ + SCI group (*p* > 0.01) (Figure 3). The beneficial effects of EP in promoting functional recovery following SCI were significantly reduced by siRNA-HSP 72 (*p* < 0.05) (Figure 2) but not by siRNA-vector (*p* > 0.05) (Figure 2).

### 2.3. The Volume of Gray Matter Contusions in the Injured Spinal Cords Was Significantly Smaller in the EP^+^ Groups

**Figure 4:**
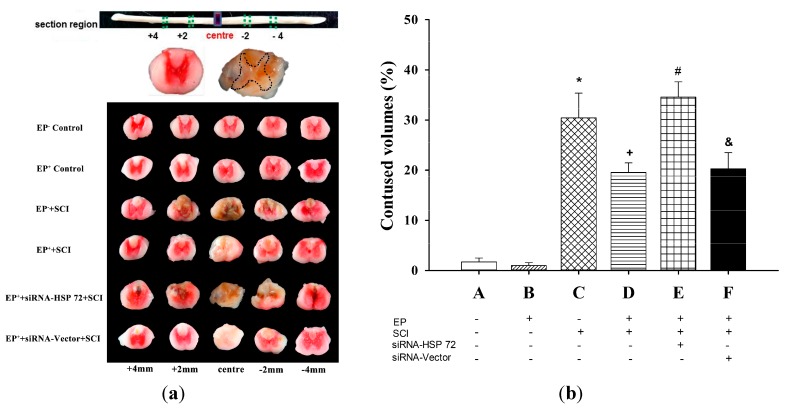
Effect of exercise on spinal cord contused volume following SCI. Contused volume was revealed by negative TTC stains and calculated as 2 mm (thickness of the slice) × (sum of the contused area in all spinal cord slices [mm^2^] [20]. The contused volumes of five sections from each spinal cord were calculated and summed 7 days after injury or sham operation. Please see the legends of Figure 1 for the abbreviations. The TTC stains presented are representative of results from one EP^−^ control, EP^+^ control, one EP^−^ + SCI, one EP^+^ + SCI, one EP^+^ + siRNA-HSP 72 + SCI + one EP^+^ + siRNA-Vector + SCI (**a**) and Values represent mean ± SD of 8 rats per group (**b**). * *p* < 0.01 for C *vs.* A or B; ^+^ *p* < 0.05 for D *vs.* C; ^#^ *p* < 0.05 E *vs.* D; ^&^ *p* < 0.05 for F *vs.* E. Please see the legends of Figure 1 for the explanations of the abbreviations.

To examine the volume of contused gray matter, serial 10-µm longitudinal sections through the lesion site were selected and used for 2,3,5-triphenyltetrazolium chloride (TTC) staining [20]. The contused volume of gray meter from five sections (4 mm rostral to the lesion site, 2 mm rostral to the lesion site, the lesion site, 2 mm caudal to the lesion, and 4 mm caudal to the lesion site) were calculated and summed 7 days post-laminectomy (Figure 4a). Rats in the EP^+^ + SCI group had significantly less contused gray matter than did rats in the EP^−^ + SCI group (*p* < 0.05 for D *vs.* C) (Figure 4b), and rats in the EP^+^ + siRNA-vector + SCI group (*p* > 0.05 for D *vs.* E) (Figure 4b). Rats in EP^+^ + siRNA-HSP 72 + SCI group had significantly less contused gray matter than did rats in the EP^+^ + siRNA-vector group (*p* < 0.05 for E *vs.* D) (Figure 4b).

### 2.4. Post-Laminectomy Levels of Gray Matter Neuronal and Astroglial Apoptosis Were Significantly Lower in the EP^+^ Groups

**Figure 5:**
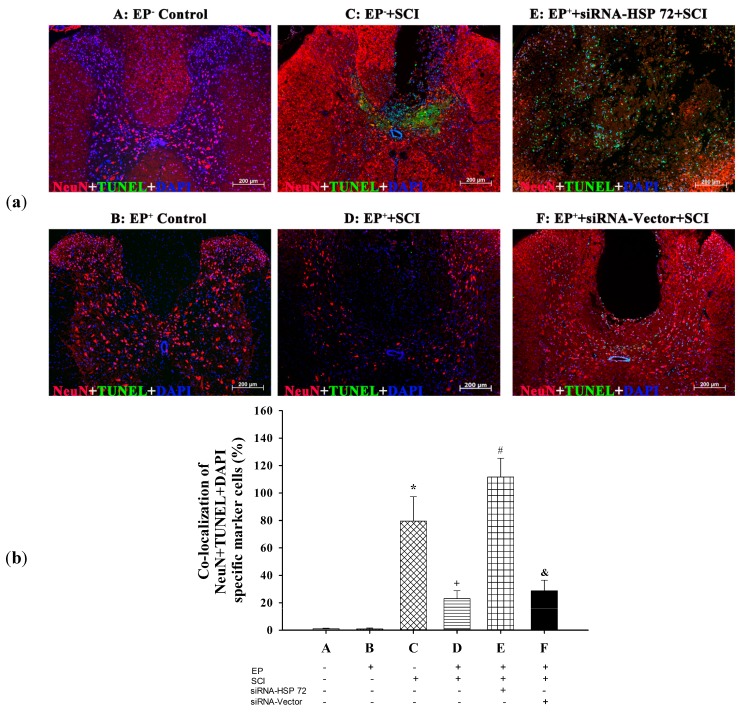
Immunofluorescence detection of apoptotic neurons and apoptotic, astrocytes in the gray matter after spinal cord injury for different groups of rats. Neuronal (**a**) and astrocytic (**b**) apoptosis was revealed by TUNEL staining. The numbers of both apoptotic neurons and apoptotic astrocytes in the lesioned sites were calculated and summed 7 days after injury or sham operation. Please see the legends of Figure 1 for the group abbreviations. The immunofluorescences presented are representative of results from different groups of rats. Values represent mean ± SD of 8 rats per group. * *p* < 0.01 for C *vs.* A or B; ^+^ *p* < 0.05 for D *vs.* C; ^#^ *p* < 0.05 for E *vs.* D; ^&^ *p* < 0.05 for F *vs.* E.

To assess the number of apoptotic cells in the gray matter in the injured spinal cord, serial longitudinal sections through the lesion site were selected and used both for immunofluorescence and for terminal deoxynucleotidyl-transferase-mediated and dUTP-biotin nick end-labeling (TUNEL) assays [21]. Neuronal and astroglial apoptosis were verified using (NeuN + TUNEL + DAPI) and (GFAP + TUNEL + DAPI) triple stains, respectively, 7 days post-laminectomy. The percentages of apoptotic neurons (Figure 5a) and apoptotic astrocytes (Figure 5b) were significantly lower in the EP^+^ + SCI group than in the EP^−^ + SCI group. Rats in EP^+^ + siRNA-HSP 72 + SCI group had significantly lower apoptotic neurons (Figure 5a) and apoptotic astrocytes (Figure 5b) than did EP^+^ + siRNA-vector + SCI group or EP^+^ + SCI group.

### 2.5. Post-Laminectomy Gray Matter Neuronal Loss Was Significantly Lower in the EP^+^ Group

**Figure 6:**
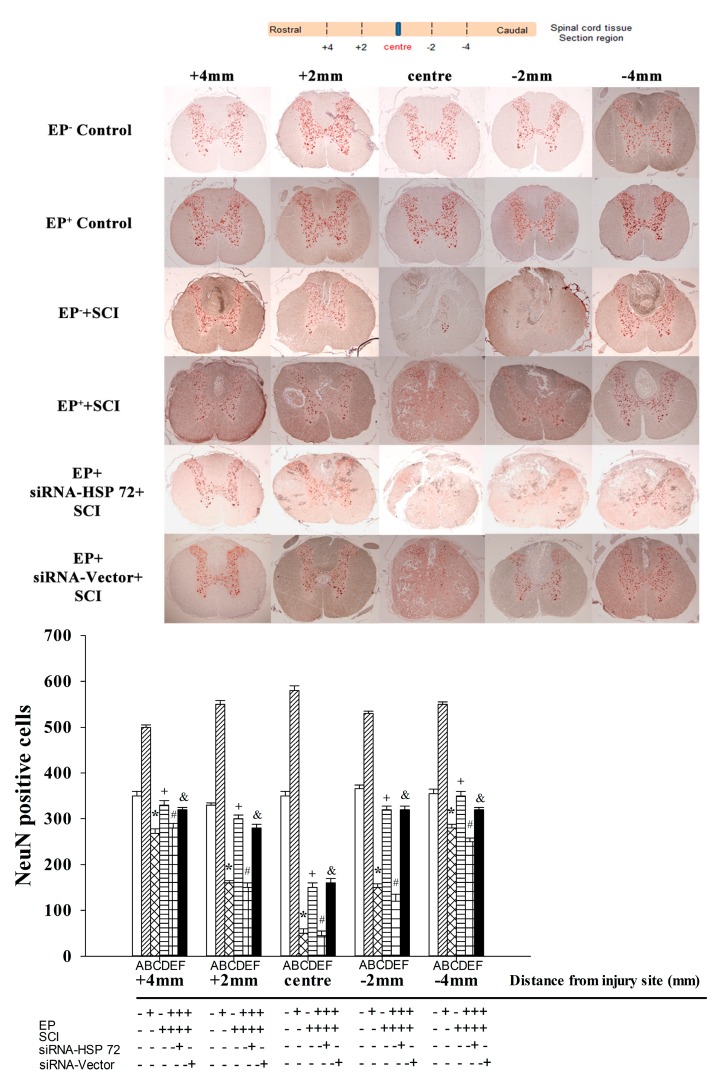
Immunohistochemical detection of neurons in the gray mater after spinal cord injury. Neurons were revealed by NeuN staining. The numbers of neurons in the gray mater from 4 mm rostral to lesioned sites to 4 mm caudal to lesioned sites were calculated and summed 7 days after injury or sham operation. Please see the legends of Figure 1 for the abbreviations. Values represent mean ± SD of 8 rats per group. The immunohistochemical stainings presented are representative of results from different groups of rats. * *p* < 0.01 for C *vs.* A or B; ^+^ *p* < 0.05 for D *vs.* C; ^#^ *p* < 0.05 for E *vs.* D; ^&^ *p* < 0.05 for F *vs.* E.

Immunohistochemical staining 7 days post-laminectomy showed that EP^+^ Control group rats had significantly (*p* < 0.01) more NeuN^+^ cells than did the EP^−^ Control group rats and that EP^+^ + SCI group rats had significantly (*p* < 0.01) more NeuN^+^ cells than did EP^−^ Control group rats and EP^+^ Control group rats (Figure 6). Rats in EP^+^ + siRNA- HSP 72 + SCI group had significantly less NeuN^+^ cells than did the EP^+^ + siRNA-vector + SCI group or the EP^+^ + SCI group (Figure 6).

### 2.6. Post-Laminectomy Underexpression of Gray Matter Neuronal and Astroglial HSP 72

**Figure 7:**
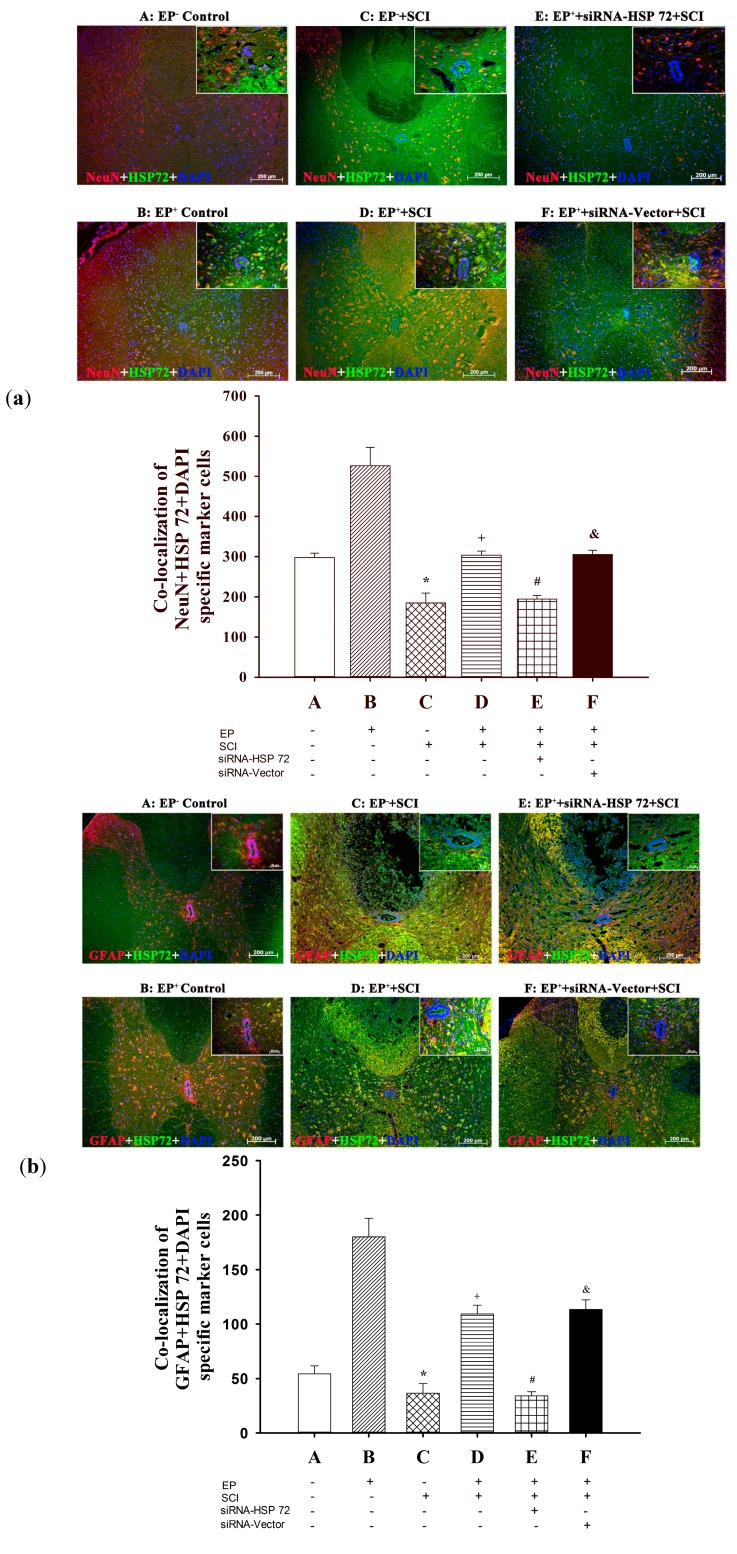
Immunofluorescence detection of HSP 72-positive neurons (**a**) and HSP 72-positive astrocytes (**b**) in the gray meter after spinal cord injury. HSP 72-positive neurons and HSP 72-positive astrocytes were revealed using double (anti-HSP 72 + anti-neuN) stains and double (anti-GFAP + anti-HSP 72) stains, respectively. The numbers of both HSP 72-positive neurons and HSP 72-positive astrocytes in the lesioned sites were calculated and summed 7 days after injury or sham operation. Please see the legends of Figure 1 for group abbreviations. The immunofluorescences presented are representative of results from different groups of animals. Values represent mean ± SD of 8 rats per group. * *p* < 0.01 for C *vs.* A or B; ^+^ *p* < 0.05 for D *vs.* C; ^#^ *p* < 0.05 for E *vs.* D; ^&^ *p* < 0.05 for F *vs.* E. The ruler for the insert image is 400×.

An immunofluorescence assay 7 days post-laminectomy was used to assess the effect of SCI-induced of neuronal and astroglial HSP 72 in the injured spinal cord. The number of NeuN + HSP 72 double-positive cells (Figure 7a) and of GFAP + HSP 72 double-positive cells (Figure 7b) was calculated and summed. EP^+^ Control group rats had significantly more NeuN + HSP 72 double-positive cells (Figure 7a) and more GFAP + HSP 72 double-positive cells (Figure 7b) than did the EP^−^ Control group rats (B *vs.* A), and the EP^+^ + SCI group rats had significantly more NeuN + HSP 72 double-positive cells (Figure 6a) and more GFAP + HSP 72 double-positive cells (Figure 7b) than did the EP^−^ + SCI group rats (D *vs.* C). Rats in EP^+^ + siRNA-HSP + SCI group had significantly lower numbers of both NeuN + HSP 72 double-positive cells (Figure 7a) and GFAP + HSP 72 double-positive cells (Figure 7b) than did the EP^+^ + SCI group or the EP^+^ + siRNA + vector + SCI group.

## 3. Discussion

Many investigators have reported that physical exercise induces cerebral HSP 72 expression in the brain, which leads to neuroprotection against heat stroke [7] and stroke [9]. The protein overexpressed in neurons reduces neuronal apoptosis by upregulating the cerebral levels of several anti-apoptotic proteins, including HSP 72 [9]. An increase in the proliferation of astrocytes has also been reported after exercise [22]. The neuroprotective effect of inducible HSP 72 expressed in astrocytes has been reported in studies of ischemic injury *in vivo* and *in vitro* [23]. The present study further showed that physical exercise induced spinal cord HSP 72. Overexpressed HSP 72 in neurons and astrocytes in the injured spinal cord reduces neuronal and astroglial apoptosis and promotes functional recovery from SCI. Earlier studies have demonstrated that neuroprotective effect was more evident on post-SCI day 7 than on day 2, suggesting the role of HSP 72 in modulating secondary injury. This study did not involve analysis of day 2.

It has been proposed that a low level of HSP 72 expression or a severe insult will block neuroprotection [24]. High levels of HSP 72 are necessary to protect the brain from denaturing stress [25,26], and HSP 72 is probably less effective in protecting against the most severe insults [25]. In the present study, exercise induced a 120% and 80% higher expression of spinal cord HSP 72 in intact rats and in SCI rats, respectively (Figure 1). Inhibiting this relatively mild expression of HSP 72 with a topical application of pSUPER plasmid expressing siRNA-HSP 72 in the injured spinal cord, which may affect the intracellular pool of neuronal and astroglial HSP 72 protein *in situ*, completely blocked exercise-induced neuroprotection. This strongly indicates that the increased neuronal and astroglial HSP 72 levels before SCI may be one of the key issues. We found that before the onset of SCI, a microinjection of siRNA into the injured spinal cord reduced neuronal and astroglial expression of HSP 72 in spinal cord tissue. We hypothesize that intravenous HSP 72 antibody [9] neutralizes HSP 72 in the spinal cord, which interferes with anti-apoptosis in SCI.

Astrocyte proliferation increases after exercise [22]. The neuroprotective effect of inducible HSP 72 expressed in astrocytes has also been reported from ischemic injury *in vivo* and *in vitro* [23]. In the present study, we found that physical exercise alone and SCI alone stimulated astrocyte proliferation in spinal cord tissue, and that a combination of physical exercise and SCI significantly increased astrocyte proliferation and astrocyte overexpression of HSP 72 in injured spinal cord tissue. Astrocytes, the predominant cell type in the human brain, are traditionally associated with contributing to increased neuroinflammation, the development of cerebral edema, and elevated intracranial pressure, all of which suggest possible roles in exacerbating secondary brain injury after a Neurotrauma [27,28,29]. In contrast, recent evidence [27,28] has indicated that astrocytes regulate brain homeostasis and limit brain injury by providing structural support within the CNS. Our findings support the essential protective role of reactive astrocytes in spinal cord injury.

It has reported that pharmacological levels of 17α-estradiol induced by conjugated estrogens vaginal cream (Premarin) protect against spinal cord apoptosis through mechanisms that involve the activation of both angiogenesis and neurogenesis [30]. Body cooling (33 °C) also improves SCI outcomes by promoting angiogenesis, neurogenesis, and anti-inflammation in a rat SCI model [31]. Hyperbaric oxygen therapy improved the outcomes of SCI by stimulating spinal cord production of both vasculoendothelial and glial cell line-derived neurotrophic growth factor and interleukin (IL)-10 (an anti-inflammatory cytokine) in rats [32].

In the present study, we have shown that exercise attenuated neuronal and astroglial apoptosis or injury in SCI by upregulating neuronal and astroglial HSP 72. Our results support the notion that HSP 72-mediated exercise preconditioning protects against SCI, at least in rats. In addition, we have developed a new procedure involving a single intraspinal injection of pSUPER plasmid expressing siRNA-HSP 72 to induce neuron-specific and astroglia-specific HSP 72 protein-knockdown in the spinal cord. The method provides a hint about its potential clinical utility as a tool for preventing SCI and other neurodegenerative disorders. The current data provide a promising treatment strategy of physical exercise preconditioning. Physical exercise preconditioning is the notion that a physical stress with a subtonic is applied to a tissue incurring a response in that tissue that then protects it from, or limits the damage when a similar, or even worse, an otherwise lethal stimulus would follow. For example, preinduction of HSP 72 in the brain and/or spinal cord tissue by physical exercise preconditioning affords protection, or tolerance of a later SCI event that would typically be devastating to the brain and/or spinal cord. The heat shock response and the induction of HSPs have paradoxical effects against cell injury. HSP induction before a pro-inflammatory stimulus is beneficial but HSP induction after a pro-inflammatory stimulus is cytotoxic. These paradoxical and contradictory effects may result from the different functions of intracellular *versus* extracellular HSPS [33]. In the present study, HSP induction is beneficial since it is induced before a pro-inflammatory stimulus. However, it was not clear as the source of HSP 72, intracellular *vs.* extracellular, or both and the origin of cellular release *i.e.*, microglial, ependymal cells or motor neurons. 

It should be stressed that our present data strongly indicate that levels of HSP 72 were correlative with extent of injury or neuroprotective effect. (Please see both Figure 3 and Figure 5). This raises the question of direct effect of HSP 72 *vs.* downstream signaling involving NFκB or entirely different neuroprotective effect by different mechanism such as upregulation of micro RNAs from exercise. A recent study demonstrated down regulation of HSP 90 ab1, HSP a4 and HSP e1 in acute SCI [34]. These findings suggest the involvement of other HSPs in SCI besides HSP 72. Finally, it should be stated that the conclusion of stimulating neurogenesis by exercise preconditioning was not supported by the present data as lower neuronal loss is not equivalent to neurogenesis and may be the result of neuroprotective effect instead.

## 4. Experimental Section

### 4.1. Ethics Statement

This study strictly adhered to the recommendations in the Guide for the Care and Use of Laboratory Animals of the National Science Council of the Republic of China. The protocol was approved by the Chi Mei Medical Center Institutional Review Board for Animal Care and Use (Assurance Number: 100120751). All efforts were made to minimize the suffering of the experimental rats.

### 4.2. Animals and Surgery

Adult male Sprague-Dawley rats weighing 254 ± 10 g were obtained from the Animal Resource Center of the Taiwan National Science Council (Taipei). They were housed in groups of four at an ambient temperature of 22 ± 1 °C with a 12-h light-dark cycle. Pellet rat chow and tap water were available *ad libitum*. The rats were first anesthetized with an intramuscular (i.m.) injection of sodium pentobarbital (25 mg/kg IP) (Sigma-Aldrich Chemical Co., St. Louis, MO, USA), and a mixture containing ketamine (44 mg/kg) (Nankang Pharmaceutical Co., Taipei, Taiwan), atropine (0.02633 mg/kg) (Sintong Chemical Industrial Co., Taoyuan, Taiwan), and xylazine (6177 mg/kg) (Bayer, Leverkusen, Germany). They then underwent a laminectomy during which the T8 and T9 vertebral peduncles were removed. The jaws of a calibrated aneurysm clip with a closing pressure of 55 g were placed on the dorsal and ventral surfaces of the spinal cord and left in place for 1 min [35]. The control rats underwent the same laminectomy, but they did not undergo compression. Rats with SCI were individually housed on special bedding to prevent skin breakdown, and their bowels and bladders were manually expressed twice daily. Food and water were freely accessible at a lowered height in their cages.

### 4.3. Recombinant pSUPER Plasmid Expressing HSP 72 siRNA Construction

pSUPER vector (Oligo Engine, Seattle, WA, USA), which contains polymerase-III H1-RNA gene promoter, can direct the synthesis of siRNA-like transcripts. The target sequence for HSP 72 (Gen Bank Accession NO. NM-0319712) was chemically synthesized (Tri-1 Biotech; Taipei, Taiwan) as complementary oligonucleotides [18]. A BLAST [36] search of the human genome database was done to ensure that the sequence did not target other gene transcripts. The synthetic oligonucleotide sip HSP 72:

5'-GATCCCCGGAGATCATCGCCAACGACTCAA GAGAGTCGTTGGCGATGATCTCCTTTTTGGAAA-3' and 3'-GGGCCTCTAGTAGCGGTTGCTGAAGTTCTCTCAG
CAACCGCTACTAGAGGAAAAACCTTTTCGAA-5'

was annealed and cloned downstream of H1 promoter to construct recombinant pSUPER/sip HSP 72 plasmid. The cloned HSP 72 target sequence was sequence-confirmed using a DNA sequencer (ABI Prism 377; Applied Biosystems, Foster City, CA, USA).

During spinal cord surgery, an acute dose of pSUPER plasmid expression HSP 72 siRNA (siRNA-HSP 72) (5 μg/rat) in 5 µL of pSUPER RNAί delivery media (siRNA-vector) was injected into the injured spinal cord at 0.5 µL/min using a microinfusion pump (type ESP-32; Eicom Corporation, Kyoto, Japan) and a 10-µL microsyringe (Hamilton Company, Tokyo, Japan). After the infusion was complete, the cannula was left in place for 5 min and then removed at 1 mm/min.

### 4.4. Exercise Training Protocol

The exercise training protocol was implemented according to the following procedures. Animals were trained on a treadmill 5 days a week for 3 weeks. Initially, animals were acclimatized to run for 15 min at 20 m/min, 0% slope for 3 days. Electrical shocks (1.0 mA) were needed initially to force animals to run forward. Subsequently, they ran without electrical shock. Then, the animals were running for 30 min at 20 m/min, 30 min at 30 m/min and 60 min at 30 m/min after 1, 2 and 3 weeks of training, respectively. No-exercise preconditioned controls were placed daily on a stationary treadmill and were given electrical stimulation in a manner identical with that used for the exercise-preconditioned group.

### 4.5. Experimental Groups and Procedures

The rats were randomly assigned to one of six groups: (1) no exercise-preconditioned controls (EP^−^ Control) [*i.e.*, no exercise preconditioning and no compression-induced SCI]; (2) exercise-preconditioned controls (EP^+^ Control) [*i.e.*, exercise preconditioning but no compression-induced SCI]; (3) no exercise preconditioning + compression-induced SCI (EP^−^ + SCI); (4) exercise preconditioning + compression-induced SCI (EP^+^ + SCI); (5) exercise preconditioning + intraspinal injection of siRNA-vector + compression-induced SCI (EP^+^ + siRNA-vector + SCI); and (6) exercise preconditioning + intraspinal injection of siRNA-HSP 72 + compression-induced SCI (EP^+^ + siRNA-HSP 72 + SCI).

In Experiment 1, the optical density (O.D.) values of HSP 72 of spinal cord were determined 7 days after the laminectomy in all groups.

In Experiment 2, the BBB scale values for all 6 groups were determined 1 day before, 1 day after, and 7 days after the laminectomy.

In Experiment 3, the percentage of the spinal cord that was contused in all 6 groups was determined 7 days after the laminectomy.

In Experiment 4, immunofluorescence staining was used to count the number of colocalizations of NeuN-, TUNEL-, and DAPI-specific markers in the spinal cord in all 6 groups 7 days after the laminectomy.

In Experiment 5, immunofluorescence staining was used to count the number of colocalization of NeuN in the spinal cord 7 days after the laminectomy.

In Experiment 6, immunofluorescence staining was used to count the number of colocalizations of NeuN-, HSP 72, and DAPI-specific markers in the spinal cord in all 6 groups 7 days after the laminectomy.

In Experiment 7, immunofluorescence staining was used to count the number of colocalizations of GFAP-, HSP 72-, and DAPI-specific markers in the spinal cord in all 6 groups 7 days after the laminectomy.

### 4.6. Assessing Hind-Limb Locomotor Function

Beginning at 24 h before injury and 7 days using the BBB open-field locomotor scale, post-SCI locomotor function was assessed both 24 h before the compression-induced injury and again 7 days later, as previously described [21]. Two trained technicians blinded to the treatments given to the rats scored their hind-limb locomotor function. The rats were observed for 4 min in an open field and scored from 0 (no observable hind-limb movements) to 21 (normal locomotion with consistent plantar stepping and coordinated gait, toe clearance, parallel paw position, trunk stability, and tail consistently in an upper position).

### 4.7. Assessing the Volume of Spinal Cord Contusions 7 Days after SCI

To examine the volume of spinal cord contusions, serial 10-µm longitudinal sections through the anterior horn were selected and stained with 2,3,5-triphenyltetrazolium chloride (TTC) at 37 °C. The volume of spinal cord contusions 7 days after SCI, revealed by negative TTC stains (pale) that indicate dehydrogenase-deficient tissue, was measured in each slice and summed using computerized planimetry [37]. The contusion volume was calculated as 2 mm (thickness of the slice) × (sum of the contused area in all spinal cord slices [mm^2^]) [19]. The contused volumes of gray matter from 5 sections (4 mm rostral to the lesion site, 2 mm rostral to the lesion site, the lesion site, 2 mm caudal to the lesion site, and 4 mm causal to the lesion site) were calculated and summed. The technicians were also blinded to the treatment group in assessing the volume of contused spinal cord.

### 4.8. Preparing the Tissue Samples

At predetermined time points after SCI, the rats were anesthetized with sodium pentobarbital, perfused, using a cardiac puncture, with 0.1 M of phosphate buffered saline (PBS) [pH 7.4] and subsequently with 4% paraformaldehyde in 0.1 M of PBS [pH 7.4]. A 20-mm section of the spinal cord, centered at the lesion site, was resected and post-fixed by immersing it in 4% paraformaldehyde overnight. The segment was then embedded in paraffin. Longitudinal sections were then cut at either 10 μm for paraffin-embedded tissues or at 8 µm for frozen tissue.

### 4.9. Protein Analysis and Quantification

Western blotting of the spinal cord tissue samples was done 7 days after SCI. The rats were overdosed with sodium pentobarbital and spinal cord samples approximately 2 mm long from the lesion epicenter (T4) and rostral (T3) and caudal (T5) segments were harvested, weighed, and immediately placed in ice-cold extraction buffer (100 mM Tris buffer, pH 7.4), 750 mM of NaCl, 2 mM of EDTA, 1% bovine serum albumin (BSA), 2% Triton X-100) in the presence of protease inhibitors (Roche) and 10 mM phenyl-methyl sulphonyl-fluoride (PMSF). Samples were sonicated and spun at 14 K for 30 min at 4 °C. Supernatants were collected and stored at −80 °C for Western blotting. The samples were boiled in standard Laemmli sample buffer for 2 min. Equal amounts of total protein were dissolved in 10% SDS-PAGE gels and transferred onto polyvinylidene difluoride (PVDF) membranes (BioRad). Each nitrocellulose replica was incubated with primary antibody and then incubated with the appropriate horseradish peroxidase (HRP)-conjugated secondary antibody. Primary rabbit polyclonal anti-HSP-70 (1:1000) (Cell Signaling) and HRP-conjugated secondary antibodies were used to detect corresponding proteins on the same membrane (Table 1). As a final step, membranes were probed with mouse monoclonal anti-actin antibody (1:5000) (AC15; Sigma-Aldrich) to confirm comparable protein loading for each lane. Immunoreactive bands were detected using an enhanced chemiluminescence (ECL) kit (Amersham Biosciences). The optical densities of immunopositive bands were determined (Gene Tools analysis software; Syngene Europe, Cambridge, UK), and values for each sample were normalized to actin. Our initial analysis, which separated the biochemical results by region (epicenter, caudal, and rostral to the lesion) found no differences in protein levels; thus, the results were calculated by combining the values for three areas for each rat. O.D. values were combined for each group, and the mean from the EP^−^ + SCI group was assigned an arbitrary unit of one. The results from the EP^+^ + SCI group were compared with the baseline values of the EP^−^ + SCI control group. The technicians were blinded to the treatment groups in assessing the volume of contused spinal cord.

**Table 1:** Western blotting in a spinal cord injury rat model.

Antibody	Antigen	Host	Company	Catalog#	Dilution
*Primary antibody*					
HSP 72	HSP 72	Mouse	Enzo	SPA-810	1:1000
β-actin	β-actin	Mouse	Santa Cruz	SC47778	1:1000
*Secondary antibody (conjugation)*					
Mouse IgG	Mouse IgG	Sheep	GE Healthcare	NA931	1:10,000
Rabbit IgG	Rabbit IgG	Donkey	GE Healthcare	NA934	1:40,000

### 4.10. Terminal Deoxynucleotidyl-Transferase-Mediated and dUTP-Biotin Nick End-Labeling (TUNEL) Staining and Immunostaining

Seven days after SCI, rat spinal cords were prepared as described above. Serial 10-μm longitudinal sections through the anterior horn were selected and used for TUNEL-staining with a TUNEL reaction mixture (Roche, Mannheim, Germany). They were reincubated in an anti-fluorescein antibody conjugated with horseradish peroxidase at 26 °C for 30 min, washed a second time, and then visualized using the avidin-biotin-peroxidase complex technique and 0.05% 3,3-diaminobenzidine-tetrachloride (Sigma-Aldrich) as a chromogen. For double labeling, antibodies (1:200) specific to neurons (Abcam, Cambridge, UK), astrocytes (Abcam), and HSP 72 (Term) were used. Nuclei were labeled with DAPI (Molecular Probes, Eugene, OR, USA). Sections were incubated overnight with primary antibodies in PBS containing 0.5% normal bovine serum at 4 °C; secondary antibodies were incubated for 1 h at room temperature. The antibodies were, sequentially, mouse monoclonal anti-NeuN (1:200) (Abcam), mouse monoclonal anti-glial fibrillary acidic protein (GFAP) antibody (1:200) (Abcam), rabbit polyclonal anti-HSP 72 antibody (1:200) (C-Term), Alexa Fluor 568-conjugated donkey anti-mouse IgG antibody (1:400) (Invitrogen), and DyLight 488-conjugated donkey anti-goat IgG antibody (1:400) (Abcam). The sections were cover-slipped with fluorescent mounting medium (Dako, Glostrup, Denmark). All the gray matter cells stained positive within an area extending between 2 mm rostral and 2 mm caudal to the lesion site were counted in two sections from each spinal cord. The labeled cells were calculated in 5 coronal sections from each rat and expressed as the mean number of cells per section. For the negative control sections from the EP^−^ Control and EP^+^ Control groups, all the procedures were done without the primary and secondary antibodies for multiple-staining (Table 2). Serial sections were also stained (1:200) with mouse monoclonal anti-NeuN neuron-specific antibody (Abcam) for histological analysis.

**Table 2:** Immunofluorescent staining in a spinal cord injury rat model.

Antibody	Antigen	Host	Company	Catalog#	Dilution
*Primary Antibody*					
NeuN	Neuron	Mouse	Millipore	MAB377	1:400
GFAP	Astrocyte	Mouse	Abcam	ab4648	1:200
TUNEL	Apoptosis		Clontech	630108	1:200
HSP 72	HSP 72	Rabbit	Abgent	AJ1380a	1:200
DAPI	Nucleic acids		Invitrogen	D1306	1:20,000
*Secondary Antibody (conjugation)*					
Mouse IgG (Alexa Fluor 568)	Mouse IgG	Goat	Invitrogen	A11031	1:200
Rabbit IgG (Alexa Fluor 488)	Rabbit IgG	Goat	Invitrogen	A11034	1:200

### 4.11. Statistical Analysis

Data are means ± standard deviation (SD). Statistical analysis was done using one-way analysis of variance (ANOVA) with Fisher’s post hoc test. Analyses for behavioral variables used Student’s unpaired *t* test to compare variables between groups. Bonferroni analysis was then used when appropriate to determine post hoc significance at individual time points. Statistica 12.0 (StatSoft Holdings, Inc., Taiwan Branch, New Taipei City, Taiwan) was used to analyze all data. Significance was set at *p* < 0.05.

## 5. Conclusions

In conclusion, exercise preconditioning decreased neuronal loss and astrogliosis, and increased neuronal and astroglial levels of HSP 72 in the gray matter of normal spinal cord tissue, thereby promoting functional recovery in rats after SCI by upregulation of neuronal and astroglial HSP 72 in the gray matter of the injured spinal cord. 

## Abbreviations

SCI: spinal cord injury
HSP: heat shock protein
EP: exercise preconditioning
siRNAs: small interfering RNAs
TTC: Triphenyltetrazolium chloride
TUNEL: terminal deoxynucleotidyl-transferase-mediated and dUTP-biotin nick-labeling
NeuN: neuronal nuclei
GFAP: glial fibrillary acidic protein
BBB: blood-brain-barrier
